# Volumetric PET/CT parameters predict local response of head and neck squamous cell carcinoma to chemoradiotherapy

**DOI:** 10.1002/cam4.295

**Published:** 2014-07-10

**Authors:** Atsushi Hanamoto, Mitsuaki Tatsumi, Yukinori Takenaka, Toshimitsu Hamasaki, Toshimichi Yasui, Susumu Nakahara, Yoshifumi Yamamoto, Yuji Seo, Fumiaki Isohashi, Kazuhiko Ogawa, Jun Hatazawa, Hidenori Inohara

**Affiliations:** 1 Department of Otorhinolaryngology—Head and Neck Surgery, Osaka University Graduate School of MedicineOsaka, Japan; 2 Department of Nuclear Medicine and Tracer Kinetics, Osaka University Graduate School of MedicineOsaka, Japan; 3 Department of Radiology, Osaka University Graduate School of MedicineOsaka, Japan; 4 Department of Biomedical Statistics, Osaka University Graduate School of MedicineOsaka, Japan; 5 Department of Radiation Oncology, Osaka University Graduate School of MedicineOsaka, Japan

**Keywords:** Chemoradiotherapy, head and neck squamous cell carcinoma, local response, metabolic tumor volume, total lesion glycolysis

## Abstract

It is not well established whether pretreatment ^18^F-FDG PET/CT can predict local response of head and neck squamous cell carcinoma (HNSCC) to chemoradiotherapy (CRT). We examined 118 patients: 11 with nasopharyngeal cancer (NPC), 30 with oropharyngeal cancer (OPC), and 77 with laryngohypopharyngeal cancer (LHC) who had completed CRT. PET/CT parameters of primary tumor, including metabolic tumor volume (MTV), total lesion glycolysis (TLG), and maximum and mean standardized uptake value (SUV_max_ and SUV_mean_), were correlated with local response, according to primary site and human papillomavirus (HPV) status. Receiver-operating characteristic analyses were made to access predictive values of the PET/CT parameters, while logistic regression analyses were used to identify independent predictors. Area under the curve (AUC) of the PET/CT parameters ranged from 0.53 to 0.63 in NPC and from 0.50 to 0.54 in OPC. HPV-negative OPC showed AUC ranging from 0.51 to 0.58, while all of HPV-positive OPCs showed complete response. In contrast, AUC ranged from 0.71 to 0.90 in LHC. Moreover, AUCs of MTV and TLG were significantly higher than those of SUV_max_ and SUV_mean_ (*P* < 0.01). After multivariate analysis, high MTV >25.0 mL and high TLG >144.8 g remained as independent, significant predictors of incomplete response compared with low MTV (odds ratio [OR], 13.4; 95% confidence interval [CI], 2.5–72.9; *P* = 0.003) and low TLG (OR, 12.8; 95% CI, 2.4–67.9; *P* = 0.003), respectively. In conclusion, predictive efficacy of pretreatment ^18^F-FDG PET/CT varies with different primary sites and chosen parameters. Local response of LHC is highly predictable by volume-based PET/CT parameters.

## Introduction

Head and neck squamous cell carcinoma (HNSCC), which includes a variety of primary sites in the upper aerodigestive tract, is a heterogeneous entity. The majority of HNSCCs are caused by tobacco and alcohol abuse, while Epstein–Barr virus (EBV) and human papillomavirus (HPV) are linked to the pathogenesis of nasopharyngeal cancer (NPC) and a subset of oropharyngeal cancer (OPC), respectively 1. Radiosensitivity and chemosensitivity vary widely, depending on the primary site and viral status, resulting in diverse clinical outcomes. OPC is one example. HPV-positive OPC responds better to radiotherapy and chemotherapy, and carries a better prognosis than HPV-negative OPC 2. Cancers of the larynx and hypopharynx constitute a subgroup of HNSCC that have overlapping clinical management strategies and share the treatment goal of larynx preservation 3. It is evident that HNSCC needs to be managed individually according to the primary site and viral status, rather than as a whole.

Chemoradiotherapy (CRT) is one of the treatment options for locally advanced HNSCC. The standard regimen is high-dose cisplatin concurrent with radiation 4. This is often associated with severe late adverse effects such as dysphagia 5. In an attempt to develop a regimen with less morbidity and equal efficacy, we conducted a phase I study of low-dose docetaxel plus cisplatin combined with radiation to determine an optimal dose of the chemotherapeutic reagents for a phase II study 6. The phase II study has been successfully finished, the results of which will be reported elsewhere. Currently this regimen of CRT is used in clinical practice in our institution. One of the most concerning issues in CRT is the difficulty and low success rate of salvage surgery for residual or recurrent disease when CRT fails 7. A solution to this issue would be pretreatment risk stratification of patients into good and poor response groups, which would lead to individualized treatment, where the poor response group would be initially treated with surgery instead of CRT. Unfortunately, classic parameters such as TNM classification are not useful for the prediction of response 8, and establishment of useful effective pretreatment risk stratification parameters is vital.

Tumor metabolic activity, measured by ^18^F-FDG PET/CT, has the potential to aid in predicting the clinical outcome after CRT in individual patients. The most commonly used ^18^F-FDG PET/CT parameter is maximum standardized uptake value (SUV_max_), which measures the highest intensity of ^18^F-FDG uptake within a region of interest (ROI). Volumetric parameters, such as metabolic tumor volume (MTV) and total lesion glycolysis (TLG), are expected to be better predictors of clinical outcome than SUV_max_. Prognostic significance of pretreatment MTV and TLG in HNSCC has been established as recently reviewed by Van de Wiele et al. 9, whereas it remains unclear whether the risk-based individualized treatment according to MTV or TLG is feasible. The accuracy of MTV and TLG in dividing patients into low- and high-risk groups has not been completely elucidated. This may be, at least in part, because of the heterogeneity of analyzed populations, involving various primary sites 10–12, different viral status 10–14, and/or various treatment modalities at different intensities 10–15.

We sought to address whether stratification of patients by pretreatment PET/CT parameters enable effective risk stratification. As the initial step, we designed the present study to elucidate which primary sites (the nasopharynx, oropharynx, or laryngohypopharynx) are evaluable by pretreatment PET/CT for prediction of local response to CRT, and which PET/CT parameter is the best predictor. To this end, we analyzed patients who had completed CRT with low-dose docetaxel plus cisplatin, and correlated local response with pretreatment PET/CT parameters in each primary site group.

## Materials and Methods

### Patients and treatment

A consecutive series of 190 patients with previously untreated HNSCC: 16 with NPC, 55 with OPC, and 119 with laryngohypopharyngeal cancer (LHC) who had undergone pretreatment ^18^F-FDG PET/CT followed by CRT in our institution between July 2007 and December 2012 were assessed. Patients were treated with conventional radiotherapy techniques (two-dimensional or three-dimensional planning and delivery). The radiation dose administered to primary tumor and involved lymph nodes was 66 Gy at fractions of 2 Gy/day, 5 days/week for OPC and LHC, and 70.2 Gy at fractions of 1.8 Gy/day, 5 days/week for NPC. The initial large radiation portals encompassed the primary tumor and entire cervical lymph node stations with 4 MV photons. The treatment fields were reduced at 40 Gy to include gross tumor volumes with adequate margins. We used the second boost fields with reduced margins typically after 56 Gy. Electrons were also used to treat the involved lymph nodes in some patients. Concurrent chemotherapy of docetaxel 10 mg/m^2^ followed by cisplatin 20 mg/m^2^ was delivered once weekly on the same day for six cycles, and was to be given before radiotherapy 6.

Pretreatment PET/CT was included in a routine work-up of HNSCC. Exclusion criteria were tracheotomy prior to PET/CT; T1 disease; a duration of greater than 6 weeks between PET/CT and the initiation of CRT; and less than 66 Gy of radiotherapy and/or fewer than five cycles of chemotherapy. Five patients who had undergone tracheotomy prior to PET/CT were excluded because tracheotomy possibly affects FDG uptake of primary tumor. Eleven patients with T1 diseases were excluded because FDG uptake is underestimated due to partial volume effect 16. Eight patients were excluded because of the duration from PET/CT to the initiation of CRT were greater than 6 weeks. Forty-eight patients who had not completed CRT were excluded to identify PET/CT parameter predictive of local response when treated at the same intensity. After application of exclusion criteria, 118 patients: 11 with NPC, 30 with OPC, and 77 with LHC were included in the study.

For response evaluation, contrast-enhanced CT and MRI were scheduled 10 weeks after the completion of CRT, while examination by direct laryngoscopy and/or endoscopy was performed 11 weeks post-CRT. Clinical and radiographic tumor responses were assessed according to Response Evaluation Criteria in Solid Tumors (RECIST version1.1) 17, and the lesser response was adopted. Detection and typing of HPV DNA in biopsy specimens of OPC was made by PCR followed by direct sequencing as reported previously 18. This retrospective study was approved by the Institutional Review Board. Written informed consent (IC) for HPV analysis was obtained from each patient, while IC for PET/CT analysis was not required.

### ^18^F-FDG PET/CT and parameters

Patients fasted for at least 4 h before the intravenous administration of approximately 3.7 MBq/kg of FDG. ^18^F-FDG PET/CT scans were performed 1 h after FDG injection by means of a dedicated scanner with 32 rings of bismuth germanate detectors that simultaneously produced 63 slices of 3.125 mm thickness along a 20 cm longitudinal field (Gemini GXL; Philips, Eindhoven, the Netherlands). All emission data were corrected for tissue attenuation by using data from the transmission scan with an external source of ^68^Ge-^68^Ga. The intrinsic resolution was 3.7 mm full width at half-maximum, and the sensitivity of the device was 7.3 cps/Bq cm^−3^. Whole-body scans were acquired in four bed positions, and were reconstructed using an iterative median root reconstruction algorithm. High-resolution transaxial, coronal, sagittal, and maximal intensity projection images were displayed on a linear gray scale monitor.

**Figure 1:**
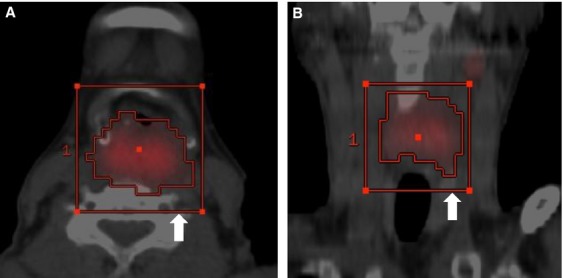
Measuring PET/CT parameters. Pretreatment transaxial (A) and coronal (B) PET/CT images of 64-year-old male with cT4aN2c hypopharyngeal cancer. The region of interest (a rectangular parallelepiped; white arrow) was set to include the primary tumor, and the SUV_max_, SUV_mean_, metabolic tumor volume (MTV), and total lesion glycolysis (TLG) were calculated automatically with the set of SUV threshold. In this case, PET/CT parameters were as follows: SUV_max_ = 15.5, SUV_mean_ = 6.0, MTV = 40.9 mL, and TLG = 245.5 g. SUV, standardized uptake value.

^18^F-FDG PET/CT data were transferred into the workstation in the digital imaging and communications in medicine format. PET/CT parameters were measured from attenuation-corrected PET/CT data using a SUV-based automated contouring program (AW suite ver. 2.0 6.5 1z; GE Healthcare, Buckinghamshire, England), which provided an automatically delineated ROI (Fig.1). The boundary was drawn large enough to incorporate a target lesion in the three imaging planes. To define the margin around the primary tumor, an SUV cutoff of 2.5 was used as previously reported 19. SUV_max_ (maximum voxel intensity within the volumetric region), SUV_mean_ (average voxel intensity), MTV, and TLG for primary tumor were calculated. MTV was defined as tumor volume with SUV over 2.5, and TLG was calculated as the product of MTV and SUVmean 20.

### Statistical analysis

The values of the PET/CT parameters in complete and incomplete responders were compared using the Wilcoxon rank sum test. Receiver-operating characteristic (ROC) analyses were made to assess the utility of PET/CT parameters to predict local response, with complete response (CR) as the gold standard. Optimal cutoff values were identified by determining the values where the sum of sensitivity and specificity was maximal. The method developed by DeLong et al. 21 was used to examine differences in the area under the curve (AUC). Univariate and multivariate analyses were made by a logistic regression model to identify independent predictors of local incomplete response. We considered the primary site, tumor stage, age, and each of the PET/CT parameters for multivariate analyses. Akaike's information criterion 22 was used to evaluate the relative usefulness of the model. All statistical analyses were performed using SAS for Windows version 9.3 (SAS Institute, Cary, NC). Two-tailed *P* < 0.05 were considered statistically significant.

## Results

### Patient characteristics

Baseline characteristics of patients are summarized in Table 1. The median duration between PET/CT and the initiation of CRT was 23 days (range, 7–42 days), while the interval was less than 30 days in 79% of patients.

**Table 1:** Baseline characteristics of patients

Factor	Level	Nasopharynx	Oropharynx	Larynx + hypopharynx
Gender	Male	8 (73%)	27 (90%)	73 (95%)
	Female	3 (27%)	3 (10%)	4 (5%)
Age	Range	48–61	37–76	42–79
	Median	55	65	65
Tumor stage	T2	4 (36%)	15 (50%)	30 (39%)
	T3	3 (28%)	6 (20%)	32 (42%)
	T4	4 (36%)	9 (30%)	15 (19%)
HPV status	Negative	–	18 (60%)	–
	Positive	–	11 (37%)	–
	Unknown	–	1 (3%)	–
Chemotherapy	5 cycles	1 (9%)	0 (0%)	5 (6%)
	6 cycles	10 (91%)	30 (100%)	72 (94%)
Radiotherapy	66 Gy	7 (64%)	30 (100%)	76 (99%)
	70 Gy	4 (36%)	0 (0%)	1 (1%)
Local response	CR	6 (55%)	25 (83%)	57 (74%)
	PR	5 (45%)	5 (17%)	20 (26%)
	SD/PD	0 (0%)	0 (0%)	0 (0%)

HPV, human papillomavirus; CR, complete response; PR, partial response; SD, stable disease; PD, progressive disease.

Eleven (37%) of 30 OPCs were HPV-positive, and HPV16 accounted for all of HPV-positive tumors. One hundred twelve (95%) and six (5%) of 118 patients underwent full cycles and five cycles of chemotherapy, respectively, while 113 (96%) and five (4%) patients received radiotherapy at a total dose of 66 Gy and 70 Gy, respectively. Eighty-eight (75%) patients showed local CR, while the remaining 30 (25%) showed partial response (PR). There were no cases of stable disease or progressive disease. No divergence was observed between clinical and radiographic responses.

### PET/CT parameters and local response according to primary site

**Figure 2:**
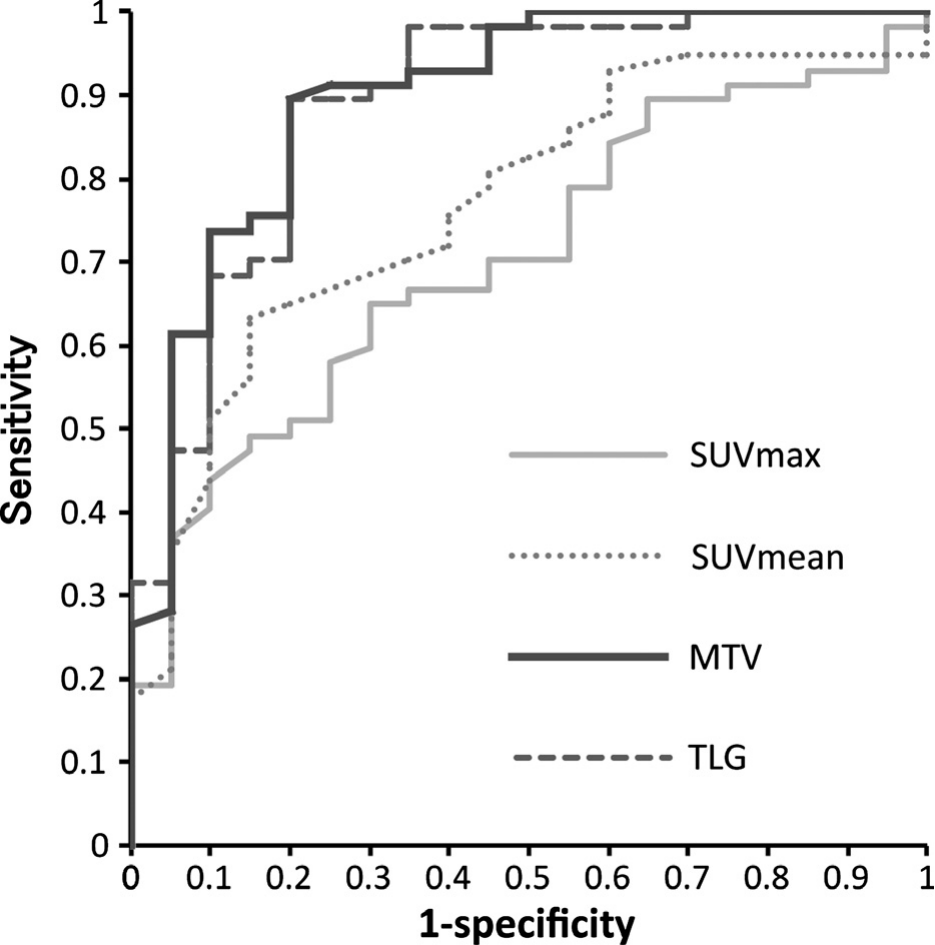
Receiver-operating characteristic curve to detect complete response in laryngohypopharyngeal carcinoma.

Table 2 summarizes the values of PET/CT parameters in complete and partial responders according to primary site and HPV status. In LHC, there was a significant difference between complete and partial responders, throughout PET/CT parameters. In contrast, no PET/CT parameter showed a difference between the two in NPC and OPC. ROC analyses were made to evaluate the usefulness of each PET/CT parameter in predicting local response, with CR as the gold standard (Fig.2). Table 3 depicts the summary of AUCs of the ROC curve according to primary site. In NPC, AUC ranged from 0.53 to 0.63, indicating low accuracy of any PET/CT parameter to discriminate between complete and partial responders. In OPC, AUC ranged from 0.50 to 0.54, again indicating low accuracy. When stratified by HPV status, HPV-negative OPC showed AUC ranging from 0.51 to 0.58, while all of HPV-positive OPCs showed CR. In LHC, AUC ranged from 0.71 to 0.90, corresponding to moderate-to-high accuracy. Noteworthy are the differences in AUC between PET/CT parameters in LHC. The AUC of MTV was significantly higher than that of SUV_max_ (*P* = 0.0002) and SUV_mean_ (*P* = 0.006). Likewise, the AUC of TLG was significantly higher than that of SUV_max_ (*P* = 0.0001) and SUV_mean_ (*P* = 0.002). There was no difference in AUC between MTV and TLG (*P* = 0.44), while the difference between SUV_max_ and SUV_mean_ was significant (*P* = 0.01). These results clearly demonstrate the predictive advantage of MTV and TLG over SUV_max_ and SUV_mean_ in LHC.

**Table 2:** PET/CT parameters of responders and nonresponders according to primary site

Site	Parameter	Complete response	Partial response	*P* value
		Median (range)	Median (range)	
Nasopharynx	SUV_max_	9.4 (4.6–14.3)	9.2 (6.7–20.1)	0.93
	SUV_mean_	4.7 (3.2–6.9)	4.2 (3.8–6.2)	0.93
	MTV	37.5 (11.7–126.0)	41.5 (29.4–111.0)	0.52
	TLG	173.2 (37.3–869.4)	203.5 (111.6–688.2)	0.52
Oropharynx	SUV_max_	10.6 (5.7–23.2)	9.8 (5.9–13.4)	1.00
	SUV_mean_	4.6 (3.3–6.7)	4.5 (3.3–6.0)	0.80
	MTV	29.0 (5.5–102.0)	29.4 (11.1–65.0)	0.87
	TLG	152.2 (18.7–663.0)	147.6 (44.4–389.8)	0.76
HPV-negative oropharynx	SUV_max_	11.0 (7.1–23.2)	9.8 (5.9–13.4)	0.92
	SUV_mean_	4.7 (3.5–6.7)	4.5 (3.3–6.0)	0.66
	MTV	31.5 (5.8–102.0)	29.4 (11.1–65.0)	0.92
	TLG	174.4 (20.2–663.0)	147.6 (44.4–389.8)	1.00
HPV-positive oropharynx	SUV_max_	10.5 (5.7–14.6)	–	–
	SUV_mean_	4.5 (3.3–5.6)	–	–
	MTV	27.9 (5.5–62.1)	–	–
	TLG	136.4 (18.7–323.1)	–	–
Larynx + hypopharynx	SUV_max_	10.0 (3.7–32.0)	12.6 (5.8–21.9)	0.007
	SUV_mean_	4.5 (3.0–8.8)	5.4 (3.5–6.6)	0.0004
	MTV	12.2 (0.7–37.1)	41.5 (4.3–128.0)	<0.0001
	TLG	62.6 (2.1–252.4)	235.4 (19.3–780.8)	<0.0001

MTV, metabolic tumor volume; TLG, total lesion glycolysis; HPV, human papillomavirus; SUV, standardized uptake value.

**Table 3:** Area under the curve by receiver-operating characteristic curve analysis

Site	Parameter	AUC	95% CI	*P* value
Nasopharynx	SUV_max_	0.53	0.23–0.82	0.87
	SUV_mean_	0.53	0.23–0.82	0.86
	MTV	0.63	0.31–0.89	0.49
	TLG	0.63	0.31–0.89	0.49
Oropharynx	SUV_max_	0.50	0.32–0.69	0.98
	SUV_mean_	0.54	0.35–0.72	0.83
	MTV	0.53	0.34–0.71	0.85
	TLG	0.54	0.35–0.73	0.75
HPV-negative oropharynx	SUV_max_	0.52	0.28–0.76	0.89
	SUV_mean_	0.58	0.33–0.80	0.68
	MTV	0.52	0.28–0.76	0.89
	TLG	0.51	0.27–0.75	0.96
Larynx + hypopharynx	SUV	0.71	0.59–0.80	0.001
	SUV_mean_	0.77	0.66–0.86	<0.0001
	MTV	0.90	0.80–0.95	<0.0001
	TLG	0.89	0.81–0.96	<0.0001

AUC, area under the curve; CI, confidential interval; MTV, metabolic tumor volume; TLG, total lesion glycolysis; HPV, human papillomavirus; SUV, standardized uptake value.

### Prediction of local response in laryngeal and hypopharyngeal cancer

We further addressed the predictive significance of PET/CT parameters in LHC. An optimal cutoff point of each parameter to divide patients into high- and low-risk groups was determined by ROC analysis. Univariate analysis revealed that tumors with a high value of any PET/CT parameter were at a significantly increased risk of PR, as compared with those with a low value (Table 4). Of note, tumors with high MTV or TLG were at an extremely increased risk of residual local disease (odds ratio [OR], 34.0; 95% confidence interval [CI], 9.4–154.8; *P* < 0.001). Sensitivity and specificity for CR were 89% and 80%, respectively, for both MTV and TLG, while positive and negative predictive values were 93% and 73%, respectively. Since PET/CT parameters were significantly associated with each other, each PET/CT parameter was individually incorporated into multivariate analysis along with age, primary site, and tumor stage, and four different models were constructed (Table 5). After adjustment for age, primary site, and tumor stage, MTV and TLG remained as independent, significant predictors of local response. LHCs with high MTV (>25.0 mL) or high TLG (>144.8 g) were at a higher risk of PR as compared with those with low MTV (<25.0 mL) (OR, 13.4; 95% CI, 2.5–72.9; *P* = 0.003) or low TLG (<144.8 g) (OR, 12.8; 95% CI, 2.4–67.9; *P* = 0.003), respectively. The Akaike's information criterion was 54.9 for the model involving MTV and 55.2 for the model involving TLG, indicating that MTV is a relatively better predictor than TLG.

**Table 4:** Univariate analysis for incomplete response in laryngohypopharyngeal cancer

Factor	Level	Incomplete response		Odds ratio	95% CI	*P* value
		*N*	%			
Age	≤65 (*N* = 38)	14	37	Reference		
	>65 (*N* = 39)	6	15	0.3	0.1–1.0	0.06
Site	Larynx (*N* = 26)	4	15	Reference		
	Hypopharynx (*N* = 51)	16	31	2.5	0.8–9.7	0.12
Tumor stage	T2 or T3 (*N* = 62)	7	11	Reference		
	T4 (*N* = 15)	13	87	51.1	11.3–375.8	<0.0001
SUV_max_	≤10.7 (*N* = 43)	6	14	Reference		
	>10.7 (*N* = 34)	14	41	4.3	1.5–13.8	0.009
SUV_mean_	≤4.7 (*N* = 39)	3	8	Reference		
	>4.7 (*N* = 38)	17	45	9.7	2.9–45.2	0.0009
MTV	≤25.0 (*N* = 55)	4	7	Reference		
	>25.0 (*N* = 22)	16	73	34.0	9.4–154.8	<0.0001
TLG	≤144.8 (*N* = 55)	4	7	Reference		
	>144.8 (*N* = 22)	16	73	34.0	9.4–154.8	<0.0001

CI, confidence interval; MTV, metabolic tumor volume; TLG, total lesion glycolysis; SUV, standardized uptake value.

**Table 5:** Multivariate analysis for incomplete response in laryngohypopharyngeal cancer

Factor	Level	SUV_max_ model		SUV_mean_ model		MTV model		TLG model	
		Odds ratio (95% CI)	*P* value	Odds ratio (95% CI)	*P* value	Odds ratio (95% CI)	*P* value	Odds ratio (95% CI)	*P* value
Age	≤65	Reference		Reference		Reference		Reference	
	>65	0.6 (0.1–2.8)	0.53	0.7 (0.2–3.2)	0.38	0.6 (0.1–3.1)	0.56	0.8 (0.2–4.0)	0.79
Site	Larynx	Reference		Reference		Reference		Reference	
	Hypopharynx	2.0 (0.4–9.8)	0.39	2.1 (0.4–11.1)	0.38	1.0 (0.2–6.5)	0.97	1.0 (0.2–6.4)	0.97
Tumor stage	T2 or T3	Reference		Reference		Reference		Reference	
	T4	34.5 (5.9–100.0)	0.0001	26.3 (4.3–100.0)	0.0004	13.2 (1.8–100.0)	0.01	14.5 (2.0–100.0)	0.008
SUV_max_	≤10.7	Reference							
	>10.7	2.7 (0.6–11.5)	0.18						
SUV_mean_	≤4.7			Reference					
	>4.7			3.6 (0.8–17.8)	0.11				
MTV	≤25.0					Reference			
	>25.0					13.4 (2.5–72.9)	0.003		
TLG	≤144.8							Reference	
	>144.8							12.8 (2.4–67.9)	0.003

CI, confidence interval; MTV, metabolic tumor volume; TLG, total lesion glycolysis; SUV, standardized uptake value.

## Discussion

We analyzed the efficacy of PET/CT parameters to predict local response of HNSCC treated by CRT with curative intent. We found that there was a substantial difference in the predictive value of PET/CT parameters in different primary sites, which most probably reflects the etiological and clinical heterogeneity of HNSCC. The AUC of PET/CT parameters in NPC and OPC ranged from 0.50 to 0.63, indicating low accuracy of the parameters in distinguishing complete from incomplete responders. In contrast, the AUC in LHC ranged from 0.71 to 0.90, indicating moderate-to-high accuracy. Additionally, in LHC, the AUCs of MTV and TLG were significantly higher than those of SUV_max_ and SUV_mean_. These results demonstrate that the predictive value of PET/CT varies with the primary site and the PET/CT parameters chosen, and suggest that only LHC patients may be stratified into potential complete and incomplete responder groups according to pretreatment MTV or TLG.

It is not surprising that pretreatment MTV and TLG are superior to SUV_max_ in predicting local response. SUV_max_ represents the maximum voxel value of FDG uptake in an ROI, and thus reflects the metabolic activity of a single voxel rather than the whole tumor mass. SUV_max_ is also highly susceptible to noise 16. In contrast, MTV and TLG are volumetric parameters that are likely more relevant to clinical outcome than SUV_max_. MTV measures the volume of metabolically active tumor; and TLG, the product of MTV and SUV_mean_, represents the overall amount of FDG uptake. TLG may be more accurate than MTV in risk stratification. Some reports 10,23 have demonstrated that the clinical outcomes of OPC and NPC were better predicted by TLG than by MTV. In our series of LHCs, both SUV_mean_ and MTV were higher in incomplete responders than in complete responders, suggesting a possible synergistic advantage of TLG over MTV in the prediction of local response. Contrary to expectations, however, our results showed MTV was equivalent to or, rather, slightly superior to, TLG in the prediction of local response in LHC.

Park et al. 15 recently reported the analytical results of 81 patients with LHC. They showed that LHC patients with low-MTV lesions survived longer than those with high-MTV lesions, and that MTV was an independent prognostic factor of overall survival. Although there were only 19 events (deaths), primary site and treatment strategy were adjusted in multivariate analysis using the Cox proportional hazards model. In addition, the AUC was 0.718, corresponding to moderate accuracy. This is most likely because they assessed a heterogeneous group of patients, who had been treated with a variety of modalities, including surgery and radiation. In contrast, we restricted our population to patients who had completed the same CRT regimen, which allowed us to draw the firmer conclusion that MTV and TLG are accurate predictors of local response in LHC. The AUCs of MTV and TLG were 0.90 and 0.89, respectively, which we think justifies the use of these parameters in risk stratification. We also showed, by multivariate analysis using a logistic regression model, that MTV and TLG were independent predictors of response after CRT. Further studies of the risk stratification value of these parameters in patients who have undergone surgery as their primary treatment are warranted.

The efficacy of PET/CT parameters in predicting local response was poor in OPC. Acting on the assumption that the heterogeneity of HPV status in OPC was responsible for the poor predictability, we analyzed OPC response according to HPV status. All of the HPV-positive OPCs showed CR, which precluded ROC analysis. The AUC of PET/CT parameters in HPV-negative OPC ranged from 0.51 to 0.58, corresponding to low accuracy. These results suggest that some other factor affected the predictive value of PET/CT parameters in HPV-negative OPC, although the limited number of patients precluded an adequate analysis. The oropharynx is a hypermetabolic region where FDG accumulates physiologically, creating a high background that may artificially elevate SUV.

There is a series of studies showing the prognostic significance of volumetric PET/CT parameters in OPC, but each of these studies has its shortcomings. Moon et al. 13 analyzed 69 patients with SCC of the tonsil, and showed that the TLG of the primary tumor was an independent prognostic factor for overall survival after adjustment for many clinical factors, in spite of the limited number of deaths (*n* = 7). In addition, although the patients were treated with several different modalities with significantly different intensities, adjustments were not made for treatment modality or HPV status. Similarly, Dibble et al. 24 analyzed a small number of OPC (*n* = 16) and oral cancers (*n* = 29) together, and showed that elevated MTV and TLG were independently associated with poor overall survival after adjustment for tumor stage, smoking history, age, sex, tumor grade, and SUV_max_, but without adjustment for primary tumor site, HPV status, or treatment modality. Treatment modalities in that study included surgery, CRT, radiotherapy, and no treatment. Significantly, there were only 20 events (death or progression of disease) during the follow-up period. This suggests that the prognostic significance of MTV and/or TLG is overestimated in these studies, because the limited number of events (deaths) will disturb exact multivariate analysis using the Cox proportional hazards model. The widely accepted criterion requires 10–15 events (deaths) per variable in a multivariate analysis of survival using the Cox proportional hazards model. In contrast, the study by Lim et al. 14 is noteworthy from the standpoint of multivariate analysis, because there were a sufficient number of events (deaths). They examined 176 patients with OPC, and showed that elevated MTV and TLG were independent predictors of death after adjustment for tumor stage. Unfortunately, adjustments were not made for HPV status and treatment modality, and the patients were treated with a diversity of regimens of chemo- and/or bioradiotherapy of an unstated range of intensities.

There are two studies where HPV status has been taken into account. Cheng et al. 25 analyzed 60 patients with OPC treated with platinum-based CRT, 30 of whom had died, and showed TLG to be an independent prognostic factor after adjustment for HPV status. However, the AUC stratifying the patients into those with good and poor survival was as low as 0.686, indicating low accuracy, similar to our series of OPCs. Tang et al. 12 analyzed 64 patients with p16-positive OPC who had been treated with radiotherapy combined with either cisplatin or cetuximab. They showed a significant inverse association of MTV with survival by univariate analysis, whereas they reported that cisplatin concurrent with radiotherapy was superior to cetuximab concurrent with radiotherapy 26. Collectively, it remains unclear whether MTV and TLG serve as independent predictors of survival in patients with OPC. Given that it has been established that patients with HPV-positive or p16-positive OPC survive significantly longer than patients with HPV-negative or p16-negative OPC 2, the prognostic significance of MTV and TLG in OPC needs to be addressed on a large scale, taking HPV status into account, after adjustment for other known prognostic factors.

We failed to show the usefulness of any PET/CT parameter to predict local response in NPC, although the small number of patients caused reduced power. Xie et al. 27 showed that NPC patients with low SUV_max_ lesions survived longer than those with high SUV_max_ lesions when treated with CRT, while the AUC of the ROC curve determining the cutoff value was 0.564, corresponding to low accuracy. Chan et al. 23 examined 196 patients with stage III/IV NPC treated with CRT, and showed that an elevated TLG in the primary tumor was an independent adverse predictor of overall survival in patients with NPC treated with CRT, though the hazard ratio was as low as 1.0013 in patients with high-TLG tumors, with low-TLG tumors as reference. The same group analyzed a consecutive series of 102 patients with NPC treated by either radiotherapy or cisplatin-based CRT according to clinical stage, and found that patients with high-TLG tumors were at significantly higher risk of death (hazard ratio 4.911; 95% confidence interval, 1.031–23.400) compared with patients with low-TLG tumors, after adjustment for age, sex, and clinical stage 28. This finding, however, needs to be interpreted with caution, because there were only 14 events (deaths) at the time of analysis, which would hamper correct multivariate analysis using the Cox proportional hazards model.

Taken together, it may be concluded that attempts at pretreatment PET/CT risk stratification in NPC are not sufficiently accurate to be clinically acceptable. This is, at least in part, most likely due to the nasopharynx being a region of physiologic FDG accumulation, like the oropharynx. The heterogeneity of NPC is probably also responsible. The vast majority of NPCs are EBV-positive, and EBV-positive NPC has a better prognosis than EBV-negative NPC 29. It has been recently shown that a subset of NPCs are EBV-negative but HPV-positive 30. Any difference in prognosis between EBV-positive NPC and HPV-positive NPC remains unknown.

Our study has limitations in addition to the small number of patients with NPC or OPC. MTV was defined as the total tumor volume segmented via a threshold SUV of 2.5. However, a standard threshold delineating FDG PET/CT-positive tissues for tumor volume has not been established. Abgral et al. 31 recently examined a diversity of SUV thresholds to define MTV, and found that MTV using an SUV threshold of 5.0 was the best predictor of clinical outcome. We are also investigating the optimal SUV threshold for MTV risk stratification.

In conclusion, as the initial step in assessing the feasibility of risk stratification using pretreatment PET/CT, we have established that local response to CRT is predicted by pretreatment PET/CT in LHC, but not in NPC or OPC, and that volume-based PET/CT parameters such as MTV and TLG are independent predictors in this disease entity. Given that MTV is relatively superior to TLG in predicting local response independently and that TLG is the product of MTV and SUV_mean_, we recommend MTV for further analysis. It is of special interest whether MTV serves as an independent prognostic factor of overall survival and laryngectomy-free survival in patients with LHC treated with CRT in the setting of larynx preservation. We are currently addressing this issue, which will allow us to design individualized larynx-sparing treatment strategy based on MTV.

## References

[1] Hausen H (2006). Infections causing human cancer.

[2] Ang KK, Harris J, Wheeler R, Weber R, Rosenthal DI, Nguyen-Tân PF (2010). Human papillomavirus and survival of patients with oropharyngeal cancer. N. Engl. J. Med.

[3] Lefebvre JL, Rolland F, Tesselaar M, Bardet E, Leemans CR, Geoffrois L (2009). Phase 3 randomized trial on larynx preservation comparing sequential vs alternating chemotherapy and radiotherapy. J. Natl. Cancer Inst.

[4] Pignon JP, Le Maître A, Maillard E, Bourhis J, MACH-NC Collaborative Group (2009). Meta-analysis of chemotherapy in head and neck cancer (MACH-NC): an update on 93 randomized trials and 17,346 patients. Radiother. Oncol.

[5] Machtay M, Moughan J, Trotti A, Garden AS, Weber RS, Cooper JS (2008). Factors associated with severe late toxicity after concurrent chemoradiation for locally advanced head and neck cancer: an RTOG analysis. J. Clin. Oncol.

[6] Inohara H, Inoue T, Akahani S, Yamamoto Y, Takenaka Y, Nakagawa T (2004). Concurrent chemoradiotherapy with cisplatin and docetaxel for advanced head and neck cancer: phase I study. Anticancer Res.

[7] Lefebvre JL, Pointreau Y, Rolland F, Alfonsi M, Baudoux A, Sire C (2013). Induction chemotherapy followed by either chemoradiotherapy or bioradiotherapy for larynx preservation: the TREMPLIN randomized phase II study. J. Clin. Oncol.

[8] Salesiotis AN, Cullen KJ (2000). Molecular markers predictive of response and prognosis in the patient with advanced squamous cell carcinoma of the head and neck: evolution of a model beyond TNM staging. Curr. Opin. Oncol.

[9] Van de Wiele C, Kruse V, Smeets P, Sathekge M, Maes A (2013). Predictive and prognostic value of metabolic tumor volume and total lesion glycolysis in solid tumours. Eur. J. Nucl. Med. Mol. Imaging.

[10] Chung MK, Jeong HS, Park SG, Jang JY, Son YI, Choi JY (2009). Metabolic tumor volume of [^18^F]-fluorodeoxyglucose positron emission tomography/computed tomography predicts short-term outcome to radiotherapy with or without chemotherapy in pharyngeal cancer. Clin. Cancer Res.

[11] La TH, Filion EJ, Turnbull BB, Chu JN, Lee P, Nguyen K (2009). Metabolic tumor volume predicts for recurrence and death in head-and-neck cancer. Int. J. Radiat. Oncol. Biol. Phys.

[12] Tang C, Murphy JD, Khong B, La TH, Kong C, Fischbein NJ (2012). Validation that metabolic tumor volume predicts outcome in head-and-neck cancer. Int. J. Radiat. Oncol. Biol. Phys.

[13] Moon SH, Choi JY, Lee HJ, Son YI, Baek CH, Ahn YC (2013). Prognostic value of ^18^F-FDG PET/CT in patients with squamous cell carcinoma of the tonsil: comparisons of volume-based metabolic parameters. Head Neck.

[14] Lim R, Eaton A, Lee NY, Setton J, Ohri N, Rao S (2012). ^18^F-FDG PET/CT metabolic tumor volume and total lesion glycolysis predict outcome in oropharyngeal squamous cell carcinoma. J. Nucl. Med.

[15] Park GC, Kim JS, Roh JL, Choi SH, Nam SY, Kim SY (2013). Prognostic value of metabolic tumor volume measured by ^18^F-FDG PET/CT in advanced-stage squamous cell carcinoma of the larynx and hypopharynx. Ann. Oncol.

[16] Boellaard R, Krak NC, Hoekstra OS, Lammertsma AA (2004). Effects of noise, image resolution, and ROI definition on the accuracy of standard uptake values: a simulation study. J. Nucl. Med.

[17] Eisenhauer EA, Therasse P, Bogaerts J, Schwartz LH, Sargent D, Ford R (2009). New response evaluation criteria in solid tumours: revised RECIST guideline (version 1.1). Eur. J. Cancer.

[18] Maruyama H, Yasui T, Ishikawa-Fujiwara T, Morii E, Yamamoto Y, Yoshii T (2014). Human papillomavirus and p53 mutations in head and neck squamous cell carcinoma among Japanese population. Cancer Sci.

[19] Larson SM, Erdi Y, Akhurst T, Mazumdar M, Macapinlac HA, Finn RD (1999). Tumor treatment response based on visual and quantitative changes in global tumor glycolysis using PET-FDG imaging. The visual response score and the change in total lesion glycolysis. Clin. Positron Imaging.

[20] Kao CH, Lin SC, Hsieh TC, Yen KY, Yang SN, Wang YC (2012). Use of pretreatment metabolic tumour volumes to predict the outcome of pharyngeal cancer treated by definitive radiotherapy. Eur. J. Nucl. Med. Mol. Imaging.

[21] DeLong ER, DeLong DM, Clarke-Pearson DL (1988). Comparing the areas under two or more correlated receiver operating characteristic curves: a nonparametric approach. Biometrics.

[22] Akaike H (1974). A new look at the statistical model identification. IEEE Trans. Autom. Control.

[23] Chan SC, Chang JT, Lin CY, Ng SH, Wang HM, Liao CT (2011). Clinical utility of 18F-FDG PET parameters in patients with advanced nasopharyngeal carcinoma: predictive role for different survival endpoints and impact on prognostic stratification. Nucl. Med. Commun.

[24] Dibble EH, Alvarez AC, Truong MT, Mercier G, Cook EF, Subramaniam RM (2012). ^18^F-FDG metabolic tumor volume and total glycolytic activity of oral cavity and oropharyngeal squamous cell cancer: adding value to clinical staging. J. Nucl. Med.

[25] Cheng NM, Chang JT, Huang CG, Tsan DL, Ng SH, Wang HM (2012). Prognostic value of pretreatment ^18^F-FDG PET/CT and human papillomavirus type 16 testing in locally advanced oropharyngeal squamous cell carcinoma. Eur. J. Nucl. Med. Mol. Imaging.

[26] Tang C, Chan C, Jiang W, Murphy JD, von Eyben R, Colevas AD (2014). Concurrent cetuximab versus platinum-based chemoradiation for the definitive treatment of locoregionally advanced head and neck cancer. Head Neck.

[27] Xie P, Yue JB, Fu Z, Feng R, Yu JM (2010). Prognostic value of 18F-FDG PET/CT before and after radiotherapy for locally advanced nasopharyngeal carcinoma. Ann. Oncol.

[28] Chang KP, Tsang NM, Liao CT, Hsu CL, Chung MJ, Lo CW (2012). Prognostic significance of ^18^F-FDG PET parameters and plasma Epstein-Barr virus DNA load in patients with nasopharyngeal carcinoma. J. Nucl. Med.

[29] Goto Y, Kodaira T, Fuwa N, Mizoguchi N, Nakahara R, Nomura M (2013). Alternating chemoradiotherapy in patients with nasopharyngeal cancer: prognostic factors and proposal for individualization of therapy. J. Radiat. Res.

[30] Lin Z, Khong B, Kwok S, Cao H, West RB, Le QT (2014). Human papillomavirus 16 detected in nasopharyngeal carcinomas in Caucasian Americans but not in endemic Southern Chinese patients. Head Neck.

[31] Abgral R, Keromnes N, Robin P, Le Roux PY, Bourhis D, Palard X (2014). Prognostic value of volumetric parameters measured by (18)F-FDG PET/CT in patients with head and neck squamous cell carcinoma. Eur. J. Nucl. Med. Mol. Imaging.

